# Ishophloroglucin A Isolated from *Ishige okamurae* Protects Glomerular Cells from Methylglyoxal-Induced Diacarbonyl Stress and Inhibits the Pathogenesis of Diabetic Nephropathy

**DOI:** 10.3390/md23010048

**Published:** 2025-01-20

**Authors:** Chi-Heung Cho, Min-Gyeong Kim, Bomi Ryu, Sang-Hoon Lee

**Affiliations:** 1 Division of Functional Food Research, Korea Food Research Institute, Wanju-gun 55365, Republic of Korea; chiheungcho@kfri.re.kr (C.-H.C.); 50034@kfri.re.kr (M.-G.K.); 2 Department of Food Biotechnology, University of Science and Technology, Daejeon 34113, Republic of Korea; 3 Major of Food Science and Nutrition, Pukyong National University, Busan 48513, Republic of Korea; ryu.bomi@gmail.com

**Keywords:** brown seaweed, diabetic complication, phlorotannin, methylglyoxal-derived advanced glycation end products, mouse glomerular mesangial cells

## Abstract

*Ishige okamurae* (*I. okamuare*), an edible brown alga, is rich in isophloroglucin A (IPA) phlorotannin compounds and is effective in preventing diseases, including diabetes. We evaluated its anti-glycation ability, intracellular reactive oxygen species scavenging activity, inhibitory effect on the accumulation of intracellular MGO/MGO-derived advanced glycation end products (AGE), and regulation of downstream signaling pathways related to the AGE–receptor for AGEs (RAGE) interaction. IPA (0.2, 1, and 5 μM) demonstrated anti-glycation ability by inhibiting the formation of glucose-fructose-BSA-derived AGEs by up to 54.63% compared to the untreated control, reducing the formation of irreversible cross-links between MGO-derived AGEs and collagen by 67.68% and the breaking down of existing cross-links by approximately 91% (*p* < 0.001). IPA protected cells from MGO-induced oxidative stress by inhibiting intracellular MGO accumulation (untreated cells: 1.62 μg/mL, MGO treated cells: 25.27 μg/mL, and IPA 5 μM: 11.23 μg/mL) (*p* < 0.001) and AGE generation and inhibited MGO-induced renal cell damage via the downregulation of MGO-induced RAGE protein expression (relative protein expression levels of MGO treated cells: 9.37 and IPA 5 μM:1.74) (*p* < 0.001). Overall, these results suggest that IPA has the potential to be utilized as a useful natural agent for the prevention and management of AGE-related diabetic nephropathy, owing to its strong anti-glycation activity.

## 1. Introduction

Advanced glycation end products (AGEs) are produced by the Maillard reaction, a non-enzymatic browning reaction, and are broadly classified as endogenous or exogenous, depending on their origin [1]. Endogenous AGEs are formed during complex physiological glycation processes occurring in organs, tissues, and body fluids. Under nonenzymatic conditions, the carbonyl group of the reducing sugar reacts with the amino group of the protein or lipid to form a Schiff base. The movement of the Schiff base double bond in the unstable form produces relatively stable Amadori compounds, including methylglyoxal (MGO), 3-deoxyglucosone, and 1-deoxyglucosone. Amadori compounds generate AGEs through cyclization, rearrangement, and condensation reactions with amino groups, including arginine, lysine, and cysteine [2,3]. AGEs are produced in the body by spontaneous chemical reactions, and highly reactive intermediate dicarbonyl compounds, such as MGO, are converted to D-lactate and excreted by the glyoxalase system, which functions as a detoxification system [4]. However, in patients with chronic metabolic diseases such as type 2 diabetes, the production of AGEs shows a substantial increase owing to the consistently high blood sugar levels, which increases the accumulation of AGEs in the body [1,2]. The main source of exogenous or dietary AGEs (dAGEs) is the consumption of heat-cooked foods [5]. These compounds are generated during the various processes involved in the cooking (boiling, baking, and frying) or processing (sterilization, packaging, and storage) of food [6]. An increase in the production of endogenous AGEs and the higher uptake of dAGEs results in elevated levels of oxidative stress, enhanced inflammatory responses, and increased incidence of cardiovascular disease in both healthy individuals as well as patients with chronic diseases [3,7]. Therefore, many researchers have proposed the strategy of reducing the production of AGEs and suppressing their accumulation in the body to prevent the pathogenesis of AGE-related disorders.

MGO is a highly reactive carbonyl compound that combines with lysine, arginine, and cysteine to form AGEs; these compounds include Nε-carboxy-ethyl-lysine (CEL), methylglyoxal lysine dimer (MOLD), methylglyoxal-derived hydroimidazolone (MG-H1), argpyrimidine, tetrahydropyrimidine (THP), and carboxy methyl cysteine (CMC) [8]. The blood concentration of MGO was found to be two to eight times higher in people with type 2 diabetes, compared with that in the control group [9]. Under normal conditions, the glyoxalase system, a detoxification mechanism involving glyoxalase-1 and glyoxalase-2, MGO is converted to a non-toxic compound, D-lactate, and is excreted from the body. However, in patients with type 2 diabetes, chronic hyperglycemia results in the production of excessive amounts of MGO. Their levels exceed the capacity of the detoxification system, leading to high blood levels of MGO [10]. MGO increases the production of reactive oxygen species that induce oxidative stress within cells, resulting in cell damage through protein damage and accelerated apoptosis [1,7]. In addition, high blood levels of MGO are closely related to increased protein expression of receptor for AGEs (RAGE) and an enhanced inflammatory response. An increase in the blood levels of MGO triggers a series of biological reactions, resulting in structural damage and dysfunction in the kidneys; these include an increase in the levels of the generated MGO-derived AGEs, in accumulation of AGEs in kidney tissue, the MGO-derived AGE–RAGE interaction, and RAGE protein expression. It may also involve the activation of apoptosis-related signaling pathways and a decrease in the expression of antioxidant-related enzymes [8,11,12]. Therefore, several studies have reported on various strategies for the prevention of diabetic complications caused by MGO or MGO-derived AGEs, such as activating anti-glycation reactions, scavenging MGO through MGO-trapping, and inhibiting the AGE–RAGE interaction [13,14,15,16,17,18].

Seaweed is a promising functional food and a rich source of bioactive substances, including fucoidans, polysaccharides, and phlorotannins; these phytochemicals have been shown to help in disease mitigation [19]. *Ishige okamurae* (*I. okamuare*), an edible brown alga found in the coastal area of Jeju Island, South Korea, is rich in phlorotannins, a major bioactive compound. A study on 38 types of seaweed extracts investigated their inhibitory effect on AGE production. Brown algae was found to contain more phenolic and phlorotannin compounds than green and red algae. It also showed a stronger inhibitory effect on AGE production. *I. okamurae* extract contains the highest amount of phlorotannin compounds and has been reported to act as a potent AGE inhibitor [20]. *I. okamurae* is rich in isophloroglucin A (IPA) phlorotannin compounds, which are effective in preventing various diseases, including obesity, diabetes, melanogenesis, muscle contraction, and osteoporosis [21,22,23,24,25,26]. Although a few studies have identified the mechanisms of *I. okamurae* anti-glycation activity and inhibition of diabetic nephropathy [27], there have been no reported studies to date on IPA, the main bioactive compound.

Therefore, in this study, we aimed to evaluate the anti-glycation ability of IPA by studying its effects on AGE formation, the formation of irreversible cross-links between MGO-derived AGEs and collagen, and the breakdown of existing cross-links. In order to evaluate the renoprotective effect of IPA, its effectiveness in protecting mesangial cells from MGO-induced oxidative stress, and its inhibitory effect on the intracellular accumulation of ROS/MGO/MGO-derived AGEs were measured. In addition, the effect of IPA on the expression of proteins related to RAGE, apoptosis, and nuclear factor erythroid-2-related factor 2 (Nrf2)/antioxidant response element (ARE), which are the major signaling pathways triggered by MGO-derived AGE-RAGE interactions, was measured using western blotting.

## 2. Results

### 2.1. Anti-Glycation Properties of IPA

The carbonyl groups of reducing sugars (glucose and fructose) and the amino groups of proteins undergo complex chain reactions under non-enzymatic conditions to produce Schiff bases and Amadori compounds; these yellow-brown compounds are known as AGEs [2]. Although AGEs are naturally produced through metabolic processes, the glycation reaction is accelerated in diabetic patients with chronic hyperglycemia, leading to an enhanced production of AGEs [1,2]. As AGEs preferentially bind to long-lived proteins, including collagen, and create irreversible cross-links, excessive production and accumulation of AGEs in the body leads to abnormal protein structural modifications in various organs. Moreover, accumulated AGEs disrupt extracellular matrix–matrix and matrix–cell interactions, which may cause organ dysfunction [3,4]. It is one of the main causes of diabetic complications such as neuropathy, retinopathy, and nephropathy in patients with type 2 diabetes. Hence, the key strategies for preventing the pathogenesis of AGE-related diabetic complications include the inhibition of the production and accumulation of AGEs, and the inhibition of AGE–collagen cross-link formation [7]. As such, numerous studies have been conducted to inhibit the production of AGEs, one of the main causes of diabetes complications, and aminoguanidine (AG) and alagebrium (ALT-711), a synthetic artificial compound that acts as a powerful AGE inhibitor, has been developed. AG showed a dramatic effect in suppressing AGE production even at very low concentrations, but it was not developed as a medicine because it showed many side effects in clinical trial tests [28,29,30].

**Figure 1:**
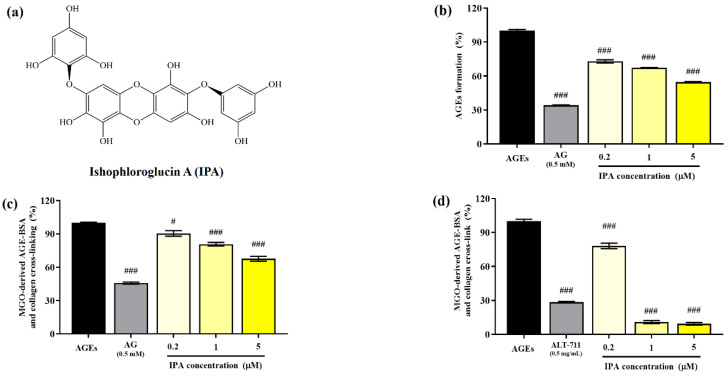
Anti-glycation abilities of isophloroglucin A (IPA). (**a**) Structure of IPA isolated from *Ishige okamurae*. (**b**) Inhibitory effect of IPA on formation of AGEs based on the glucose—BSA system. (**c**) Inhibitory effect of IPA on irreversible cross-linking between MGO-derived AGEs and collagen. (**d**) Effect of IPA on breakdown of established cross-links between MGO-derived AGEs and collagen. Aminoguanidine (AG; 0.5 mM) and alagebrium (ALT-711; 0.5 mg/mL) were used as the positive controls. Each experiment was independently performed three times (*n* = 3). All data are presented as mean ± standard deviation. Statistical analysis of the results was performed using one-way ANOVA, followed by Turkey’s test (# *p* < 0.05, and ### *p* < 0.001 vs. the untreated control).

The structure of isophloroglucin A (IPA) from *Ishige okamurae* is shown in Figure 1a. The inhibitory effect of the glucose–BSA system on AGE production was measured to evaluate the anti-glycation ability of IPA. The formation of AGEs was significantly suppressed by IPA treatment (### *p* < 0.001; Figure 1b). An increase in the concentration of IPA led to a significant increase in the inhibitory rate of generation of AGEs (### *p* < 0.001) and showed a maximum value of 54.62 ± 0.84%. AG (0.5 mM), which was used as the positive control, reduced the production of AGEs to 34.13 ± 0.58%.

The irreversible crosslinking of collagen induced by AGEs produced in the body or consumed through food results in organ dysfunction. Therefore, the inhibition of the formation of these irreversible crosslinks is an important strategy for preventing AGE-induced diabetic complications. The addition of IPA significantly reduced the formation of irreversible crosslinks between MGO-derived AGEs and collagen (# *p* < 0.05, ### *p* < 0.001; Figure 1c). The cross-link formation was suppressed to 67.68 ± 3.82% at the maximum treatment concentration of IPA (5 μM).

The effect of IPA on the disruption of the irreversible crosslinks present between MGO-derived AGEs and collagen is shown in Figure 1d. All treatment concentrations of IPA showed a significant reduction in the cross-links formed between MGO-derived AGEs and collagen (### *p* < 0.001). In particular, IPA at concentrations of 1 (10.89 ± 2.35%) and 5 μM (9.38 ± 1.95%) was more effective than the cross-link breaker ALT-711 (28.49 ± 1.05%).

Several studies have focused on the inhibition of AGE production for the treatment of AGE-related disorders, with special attention paid to plant extracts and phytochemicals. The inhibitory activity of procyanidins has been shown to depend on the structure of the compound. Catechin was found to inhibit the production of AGEs more effectively than epicatechin, which is closely related to its radical scavenging activity [16]. In addition, several researchers have suggested the possibility of sequestering intermediate reactive carbonyl species such as MGO, a precursor of AGEs, in order to inhibit AGE formation. Several phenolic and flavonoid compounds were reported to inhibit the production of MGO-derived AGEs, owing to the formation of mono- or di-MGO adducts via MGO-trapping reactions [15,31]. Here, it was demonstrated that IPA not only inhibited AGE production but also reduced the generation of cross-links between MGO-derived AGEs and collagen, thereby showing considerable promise for development as a natural AGE inhibitor.

### 2.2. Renoprotective Effects of IPA Against MGO-Induced Oxidative Stress

MGO, a highly reactive dicarbonyl compound, increases intracellular ROS levels and causes renal cell damage. MGO-induced oxidative stress results in a decrease in elasticity, structural deformation, and the dysfunction of kidney proteins; together, these factors contribute to the pathogenesis of diabetic nephropathy [11]. Therefore, a decrease in intracellular ROS levels caused by MGO-induced oxidative stress may contribute to renal cell protection.

**Figure 2:**
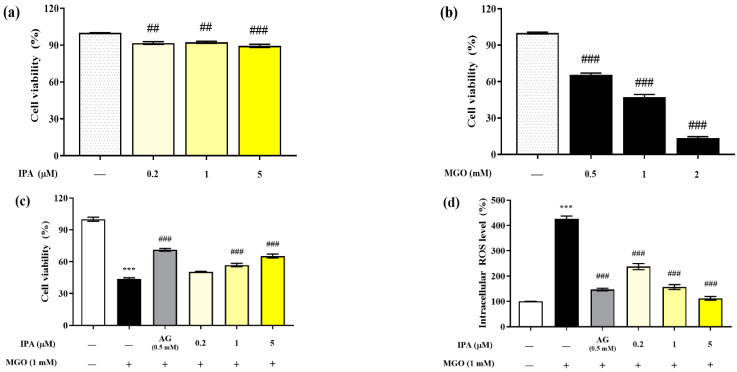
Renoprotective effects of IPA on MGO-induced oxidative stress in mesangial cells. The IPA cytotoxicity (**a**), MGO toxicity (**b**), protective effect of IPA from MGO—induced oxidative stress in mesangial cells (**c**), and intracellular ROS scavenging ability of IPA (**d**) were measured. Each experiment was independently performed six times (*n* = 6). Data are expressed as mean ± standard deviation. The statistical analysis of the results was performed using one-way ANOVA, followed by Tukey’s test (*** *p* < 0.001 vs. normal and ## *p* < 0.01, ### *p* < 0.001 vs. MGO only).

The MTT assay was performed to evaluate the cytotoxicity of IPA on mouse glomerular mesangial cells (Figure 2a). The viabilities of mesangial cells after exposure to 0.2, 1, and 5 μM of IPA for 24 h were 91.04 ± 1.91%, 92.32 ± 1.36%, and 89.47 ± 2.28%, respectively, compared with those of the untreated cells. Hence, IPA was found to be non-toxic to mesangial cells at all treatment concentrations. In addition, the treatment of mesangial cells with MGO reduced the viability in a concentration-dependent manner (Figure 2b). Treatment with 0.5 mM MGO reduced the viability of mesangial cells to 47.37 ± 4.97%. A concentration of 1 mM MGO was used in all subsequent cell experiments.

Pretreatment with IPA (1 and 5 µM) significantly increased the viability of mesangial cells by offering protection against MGO-induced oxidative stress; the viability increased to 65.38 ± 3.21% at the maximum treatment concentration of 5 μM (### *p* < 0.001) (Figure 2c). In mesangial cells exposed to MGO (1 mM) for 24 h, the intracellular ROS level increased by 427.42 ± 17.29% compared to the untreated cells (Figure 2d). In contrast, pretreatment with IPA significantly reduced the intracellular ROS levels at all concentrations (### *p* < 0.001). In particular, 1 (156.84 ± 16.76%) and 5 (111.99 ± 12.59%) μM IPA showed a similar reduction in ROS levels as AG (146.92 ± 8.33%).

Several previous studies have reported that brown algal seaweeds and phlorotannin compounds effectively protect mesangial cells from MGO-induced oxidative stress. Phlorotannin compounds, including dieckol and diphlorethohydroxycarmalol, showed similar effects on MGO-induced intracellular ROS levels as the positive control AG [17,18]. Our results demonstrate that IPA effectively protects mesangial cells from oxidative stress by serving as a scavenger of intracellular ROS induced by MGO.

### 2.3. Inhibitory Effects of IPA on the Generation of Intracellular AGEs

Compared with the control group, people with type 2 diabetes have been found to have two- to eight-fold more MGO in their bodies. An increase in plasma MGO levels may lead to organ dysfunction, with serious effects on blood vessels, eyes, liver, nerves, and kidneys. Increased MGO levels in the blood directly contribute to the progression of diabetic nephropathy by triggering progressive structural, biochemical, and functional changes in kidney disease markers [4,32,33]. Additionally, the intracellular accumulation of MGO serves as a precursor for various AGEs such as CEL, MOLD, MG-H1, argpyrimidine, THP, and CMC. These compounds cause various structural modifications and functional disorders, accompanied by oxidative stress [11]. Especially, MGO or MGO-derived AGE-induced diabetic nephropathy is characterized by the accumulation of extracellular matrix (ECM) proteins in the glomeruli and tubular interstitium, resulting in structural changes such as changes in packing density and surface charge and ECM stiffness [1,4,34]. Hence, it is important to inhibit intracellular AGE production to suppress diabetic nephropathy caused by MGO or MGO-derived AGEs. Therefore, in this study, an in vitro study was performed using mouse glomerular mesangial cells to verify the effectiveness of IPA in alleviating MGO or MGO-derived AGE-induced diabetic nephropathy.

**Figure 3:**
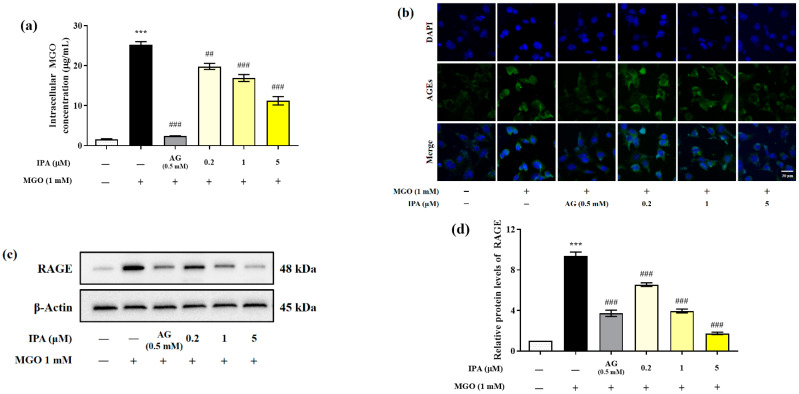
Inhibitory effects of IPA on the accumulation of intracellular MGO and MGO-derived AGEs in mesangial cells. After seeding, the mesangial cells were pretreated with IPA for 1 h. Subsequently, 1 mM MGO was added, and the cells were cultured for 24 h. The inhibitory effect of IPA on intracellular MGO accumulation was quantitatively measured using OxiSelect™ MGO Competitive ELISA Kit (OxiSelect™; Cell Biolabs Inc., San Diego, CA, USA) (**a**) and confirmed by immunofluorescence staining using AGEs antibody/DAPI double staining (**b**). The effect of IPA on RAGE protein expression in MGO—induced mesangial cells was measured using western blot (**c**). Relative RAGE band intensity; β-actin was used as an internal control (**d**). Data are expressed as mean ± standard deviation (*n* = 3). Statistical analysis of the results was performed using one-way ANOVA, followed by Tukey’s test (*** *p* < 0.001 vs. control, ## *p* < 0.01, and ### *p* < 0.001 vs. MGO only).

Quantitative analysis was performed using the OxiSelect^TM^ Methylglyoxal (MG) Competitive ELISA kit (OxiSelect™; Cell Biolabs Inc., San Diego, CA, USA) to evaluate the effect of IPA on the inhibition of intracellular MGO accumulation (Figure 3a). The intracellular MGO content of untreated mesangial cells was 1.62 ± 0.19 μg/mL. In the cells treated with MGO (1 mM) for 24 h, the intracellular MGO content rapidly increased to 25.27 ± 1.26 μg/mL (*** *p* < 0.001). In contrast, pretreatment with IPA (5 μM) significantly reduced intracellular MGO accumulation to 11.23 ± 1.81 μg/mL (## *p* <0.01, ### *p* < 0.001).

Immunofluorescence analysis was performed to further verify the inhibitory effect of IPA on the accumulatio of intracellular AGEs (Figure 3b). Treatment with MGO induced the accumulation of AGEs in mesangial cells, which was observed as an increase in the green fluorescence intensity, compared to the untreated control. However, treatment with IPA was again found to suppress the accumulation of AGEs.

MGO or MGO-derived AGEs activate downstream signaling pathways, including those for inflammation and apoptosis, through specific binding to the receptor for AGE (RAGE) protein. Western blotting was performed to evaluate the effect of IPA on the MGO-induced RAGE expression (Figure 3c). Treatment with MGO increased RAGE protein expression in mesangial cells by almost 9.3-fold compared with that in untreated cells (*** *p* < 0.001) (Figure 3d). In contrast, pretreatment with IPA significantly inhibited MGO-induced RAGE protein expression at all concentrations tested (### *p* < 0.001). In particular, 20 μM IPA (1.75-fold) suppressed RAGE protein expression more effectively than AG (3.72-fold).

An increase in the plasma and intracellular levels of MGO results in oxidative stress and inflammation, leading to the dysfunction of retinal capillary endothelial cells, human lens epithelial cells, and mesangial cells. In addition, MGO accumulation increased the expression of RAGE protein and the production of MGO-derived AGEs. The AGEs interact with RAGE proteins to activate downstream signaling pathways such as inflammation and apoptosis [35,36,37]. Polyphenol compounds such as quercetin, phloretin, and phloridzin inhibit intracellular MGO accumulation by sequestering MGO and forming adducts [38,39,40]. In addition, the extracts of *Ecklonia cava* and *Ishige okamurae*, which are brown marine algae, acted as strong MGO scavengers and inhibited its accumulation in mesangial cells [27,41]. Further, molecular docking studies have shown that dieckol and diphlorethohydroxycarmalol derived from seaweed suppress the interaction between MG-H1 and RAGE through competitive inhibition [17,18]. Our results indicated that IPA suppressed AGE-RAGE interactions by regulating RAGE protein expression through the inhibition of the accumulation of MGO and MGO-derived AGEs in mesangial cells.

### 2.4. Preventive Effects of IPA Against MGO-Induced Renal Cell Apoptosis

MGO or MGO-derived AGEs (CEL, MOLD, MG-H1, CMC, argpyrimidine, and THP) accumulate in various organs and induce apoptosis. MGO promotes apoptosis in HUVEC by increasing the Bax/Bcl-2 ratio and activating cleaved caspase-3 [42]. MGO-induced activation of p38 mitogen-activated protein kinase (MAPK) is closely related to the induction of diabetic neuropathy and nephropathy, since it induces apoptosis of Schwann and mesangial cells [43,44]. Therefore, the modulation of apoptosis-associated protein levels is considered as an important step in preventing AGE-induced diabetic nephropathy.

**Figure 4:**
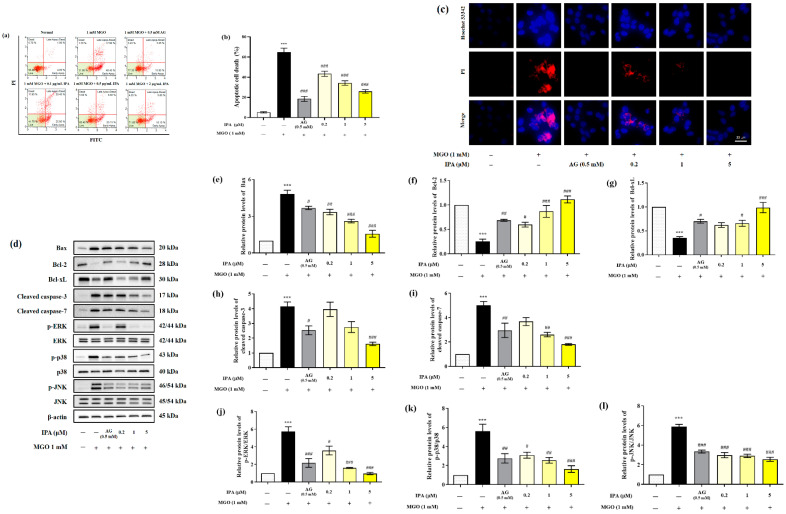
Protective effect of IPA against MGO—induced renal cell apoptosis. After seeding, mesangial cells were pretreated with IPA for 1 h. Following the addition of 1 mM MGO, the cells were further cultured for 24 h. The effect of IPA on protection from MGO—induced apoptosis in mesangial cells was measured using a MUSE cell analyzer with a Muse™ Annexin V & Dead Cell kit (Luminex, TX, USA) (**a**). The graph represents the proportion of early and late apoptotic cells (**b**). The inhibitory effect of IPA on MGO—induced apoptotic cell death was confirmed by immunofluorescence staining using Hoechst 33342/PI double staining (**c**). The effect of IPA on apoptosis-related protein expression in MGO-induced mesangial cells was measured using western blot (**d**). Relative band intensities of bax (**e**), bcl-2 (**f**), bcl-xL (**g**), cleaved caspase-3 (**h**), cleaved caspase-7 (**i**), p-ERK/ERK (**j**), p-p38/p38 (**k**), and p-JNK/JNK (**l**); β-actin was used as an internal control. Data are expressed as mean ± standard deviation (*n* = 3) and statistical analysis of the results was performed using one-way ANOVA, followed by Tukey’s test (*** *p* < 0.001 vs. control, # *p* < 0.05, ## *p* < 0.01, and ### *p* < 0.001 vs. MGO only).

The effect of IPA in inhibiting MGO-induced apoptosis was measured using a MUSE analyzer (Figure 4a). Treatment with MGO resulted in a 13-fold (65.03 ± 3.80%) increase in total apoptotic cell death (early and late apoptosis) of mesangial cells, when compared to the untreated cells (5.12 ± 0.70%) (Figure 4b). On the contrary, pretreatment with IPA (20 μM) significantly inhibited MGO-induced apoptosis, (25.91 ± 1.75%). The inhibitory effect of IPA on MGO-induced apoptosis was further verified by Hoechst 33342/PI double staining (Figure 4c). An increase in the number of apoptotic bodies (blue fluorescence) and cell death (red fluorescence) in mesangial cells was observed following treatment with MGO. Pretreatment with IPA resulted in the suppression of mesangial cell apoptosis, as well as cell death.

We measured the levels of Bax, Bcl-2, Bcl-xL, cleaved caspase-3, cleaved caspase-7, p-ERK/ERK, p-p38/p38, and p-JNK/JNK to confirm the effects of IPA on the molecular mechanisms of apoptosis (Figure 4d). Treatment with MGO increased the protein expression levels of Bax, cleaved caspase-3, and cleaved caspase-7. The phosphorylation levels of ERK, p38, and JNK increased from 4.15 to 5.89 in mesangial cells compared to untreated cells. Moreover, MGO treatment resulted in a decrease in the protein expression levels of bcl-2 and bcl-xL from 0.25 to 0.35 compared to the untreated cell (Figure 4e–l). On the contrary, pretreatment with IPA significantly regulated the expression of apoptosis-related proteins induced by MGO and inhibited apoptotic cell death in mesangial cells. *Lespedeza bicolor* has been shown to prevent the development of diabetic nephropathy by inhibiting apoptotic cell death of renal epithelial cells from methylglyoxal-induced glucotoxicity [45]. In addition, a baicalin–chrysin mixture prevented diabetic tubular injury in rat tubular epithelial cells by inhibiting apoptosis caused by MGO-mediated mitochondrial dysfunction [46]. Moreover, *Ishige okamurae* extract inhibited apoptotic cell death in mouse glomerular mesangial cells by inactivating MGO-induced MAPK phosphorylation [27]. These results demonstrate that MGO modulates the apoptosis-related molecular mechanism of mesangial cells. Additionally, IPA is thought to contribute to the prevention of kidney dysfunction by suppressing the apoptotic mechanisms of MGO-induced mesangial cells.

### 2.5. Effects of IPA on the Nrf2/ARE Signaling Pathway

**Figure 5:**
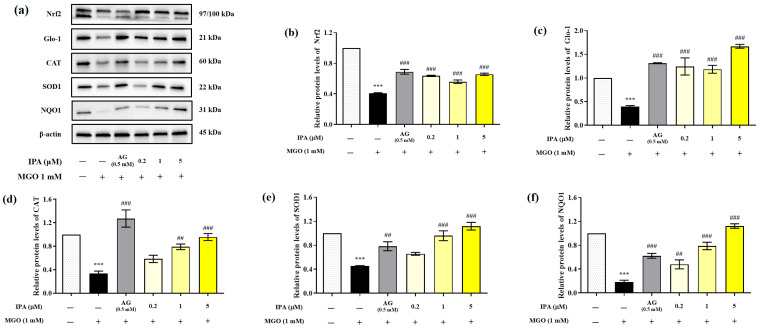
Effect of IPA on the nuclear factor erythroid-2-related factor 2 (Nrf2)/antioxidant responsive element (ARE) signaling pathway in MGO-induced mesangial cells. After seeding, mesangial cells were pretreated with IPA for 1 h, followed by the addition of 1 mM MGO, and further culturing for 24 h. The protein expression levels of Nrf2, Glo-1, CAT, SOD1, and NQO1 were measured using western blot (**a**). Relative protein expression levels of Nrf2 (**b**), Glo-1 (**c**), CAT (**d**), SOD1 (**e**), and NQO1 (**f**); β-actin was used as an internal control. Data are expressed as mean ± standard deviation (*n* = 3). Statistical analysis of the results was performed using one-way ANOVA, followed by Tukey’s test (*** *p* < 0.001 vs. control and ## *p* < 0.01, ### *p* < 0.001 vs. MGO only).

MGO significantly reduced the expression of Nrf2, Glo-1, and antioxidant enzymes (CAT, SOD1, and NQO1) in mesangial cells (*** *p* < 0.001) (Figure 5a). In contrast, pretreatment with IPA significantly suppressed the MGO-induced decrease in the protein expression levels of Nrf2, Glo-1, CAT, SOD1, and NQO1 (## *p* < 0.01, ### *p* < 0.001). IPA was found to have a strong influence on the expression of Glo-1, which contributes to the detoxification system.

## 3. Materials and Methods

### 3.1. Chemicals and Reagents

Methylglyoxal (MGO), aminoguanidine (AG), alagebrium (ALT-711), bovine serum albumin (BSA), and 3,3,5,5-tetramethylbenzidine (TMB) liquid substrate systems for the immunosorbent assay were purchased from Sigma (St. Louis, MO, USA). Collagen I-coated plates were purchased from Gibco (SPL Life Sciences, Pochen-si, Gyeonggi-do, Republic of Korea). PRO-PREP™ Protein Extraction Solution was purchased from iNtRON Biotechnology (Seongnam, Republic of Korea). The OxiSelect™ Methylglyoxal (MG) Competitive ELISA Kit was procured from Cell Biolabs Inc. (San Diego, CA, USA). Detailed information on all antibodies used in western blotting is provided in Supplementary Table S1.

### 3.2. Sample Preparation

The isophloroglucin A (IPA) was kindly donated by Professor You-Jin Jeon (Jeju National University, Republic of Korea). *Ishige okamurae* (*I. okamurae*) were collected along the coastline of Jeju Island (Seongsan), Republic of Korea, in May 2023. The collected samples were washed three times using tap water to remove salt from the surface. The washed sample was dried using a freeze dryer and stored at −20 °C. The sample (100 g) was homogenized with a grinder and extracted using 70% (*v/v*) ethanol (1 L) for 24 h while stirring at 37 °C. The *I. okamurae* 70% (*v/v*) ethanol extract was dissolved in 50% (*v/v*) methanol and then fractionated using centrifugal partition chromatography (CPC 240, Tokyo, Japan). The effluent from the column outlet was monitored with a UV detector (230 nm), and fractions were collected at 3 min intervals using a fraction collector. Fractions containing the IPA were further purified using a semipreparative HPLC column (YMC-Pack ODS-A, 10 mm × 250 mm, 5 µm). The final purified IPA was confirmed to be 99% pure [47].

### 3.3. Determination of the Inhibitory Effect on AGE Formation

The inhibitory effect on AGE formation was measured to evaluate the anti-glycation ability of IPA. The untreated AGE group was prepared using glucose (200 mM), fructose (200 mM), BSA (10 mg/mL), and 50 mM phosphate buffer (pH 7.4) in the ratio 0.5:0.5:7:2. The sample group was prepared using glucose, fructose, BSA and various concentrations of IPA (0.2, 1, and 5 μM) in the ratio 0.2:0.5:7:2. In the positive control group, the same volume of AG (0.5 mM) was added instead of IPA. Each additive was mixed and incubated in a constant temperature incubator at 37 °C for 7 days. After this period, the AGE production was quantified by measuring the fluorescence intensity at excitation (350 nm) and emission (450 nm) using a fluorescence microplate reader (Molecular Devices, Sunnyvale, CA, USA). The ability of IPA to inhibit the production of AGEs was expressed as a percentage (%) of the decrease in fluorescence intensity compared to the control group (without samples, 100%).

### 3.4. Determination of the MGO-Derived AGEs–Collagen Cross-Linking Inhibitory Ability

The anti-glycation effect of IPA was evaluated by measuring its ability to inhibit the formation of irreversible crosslinks between MGO-derived AGEs and collagen. Horseradish peroxidase (HRP)-labeled MGO-derived AGEs (5 μg/mL) and IPA (0.2, 1, and 5 μM) or AG (0.5 mM) were mixed in equal proportions. The same amount of 50 mM phosphate buffer was added instead of the sample for the untreated control group. The mixture was dispensed at 100 μL per well into a collagen-coated 96-well plate. The 96-well plate was incubated for 18 h in a constant temperature incubator at 37 °C. At the end of the incubation, the supernatant was removed, and the 96 wells were washed three times with 0.05% PBST (PBS with 0.05% Tween 20). TMB substrate solution (100 μL) was added to each well for 30 s to induce color development, and 1 N HCL solution (100 μL) was subsequently added to terminate the color development reaction. The absorbance was measured at 450 nm, and the inhibitory activity of IPA on cross-link formation was expressed as a percentage (%) of the decrease in absorbance compared to the control group (without samples, 100%).

### 3.5. Determination of the MGO-Derived AGEs–Collagen Cross-Link Breaking Ability

To evaluate the anti-glycation effect of IPA, its ability to cleave irreversible crosslinks established between MGO-derived AGEs and collagen was measured. HRP-labeled MGO-derived AGEs were dispensed into a 96-well plate and incubated in a 37 °C thermostat for 4 h to allow for the formation of cross-links. After incubation, the supernatant was removed, and 96 wells were washed three times with 0.05% PBST. IPA (0.2, 1, and 5 μM) and ALT-711 (positive control, 0.5 mg/mL) were added and allowed to react in a 37 °C constant temperature incubator for 14 h. In the untreated control group, the same amount of 50 mM phosphate buffer was added instead of the sample. The supernatant was removed and washed three times with PBST. The TMB substrate solution was added to allow color development for 30 s. After the color development reaction was terminated by adding 1 N HCL solution, the remaining crosslinks were measured by measuring the absorbance at 450 nm. The crosslinking ability of IPA was expressed as percentage (%) decrease in absorbance compared to that of the untreated control (100%).

### 3.6. Cell Culture

Mouse glomerular mesangial cells (mesangial cells) were purchased from ATCC (CRL-1927^TM^, Rockville, MD, USA) and cultured in a Dulbecco’s modified Eagle’s medium/F-12 Nutrient Mixture (DMEM/F-12; Ham, 3:1 mixture) (Welgene Inc., Daegu, Republic of Korea) containing 5% FBS, 14 mM HEPES, 100 U/mL penicillin, and 100 μg/mL streptomycin at 37 °C in 5% CO_2_ and 95% air.

### 3.7. Assessment of Cell Viability

The MTT assay was performed to evaluate the efficiency of IPA in protecting mesangial cells from MGO-induced oxidative stress. Prior to evaluating the effects of IPA, a toxicity evaluation was performed to select the appropriate treatment concentrations of IPA and MGO. Mesangial cells were distributed in a 96-well plate at a density of 2 × 10^4^/well and cultured in a constant temperature incubator at 37 °C for 4 h. To evaluate the cytotoxicity of IPA, the cells were treated with 0.2, 1, and 5 μM IPA and cultured for 24 h. To evaluate the toxicity of MGO, serum-free culture medium was treated for 1 h. Afterwards, MGO was added at concentrations of 0.5, 1, and 2 mM, and the cells were cultured for 24 h. The supernatant was subsequently removed, and MTT reagent (5 mg/mL) was added to each well and incubated for 3 h. After removing the MTT reagent, purple formazan was dissolved in 50 μL DMSO and the absorbance was measured using microplate reader at 570 nm (Infinite M200; Tecan Austria GmbH, Grödig, Austria).

To measure the effect of IPA on protecting mesangial cells from MGO-induced oxidative stress, cells were seeded in a 96-well plate (2 × 10^4^/well) and cultured for 4 h. Afterwards, the cells were pretreated with IPA (0.2, 1, and 5 μM) and AG (0.5 mM, positive control) for 1 h. MGO (1 mM) was subsequently added, and the cells were cultured for an additional 24 h. The supernatant was removed at the end of the culture period, and MTT reagent (5 mg/mL) was added to each well and incubated for 3 h. After the removal of the MTT reagent, purple formazan was dissolved in 50 μL DMSO, and the absorbance was measured using microplate reader at 570 nm (Infinite M200; Tecan Austria GmbH, Grödig, Austria). Cell viability was expressed as percentage (%) compared with the absorbance of the untreated control (without sample or MGO).

### 3.8. Intracellular ROS Production

The intracellular ROS-scavenging ability of IPA was evaluated using DCFH-DA assay. The mesangial cells were seeded in 96-well plates (2 × 10^4^/well) and incubated for 4 h. After the removal of supernatant, the cells were pre-treated with IPA (0.2, 1, and 5 μM) or AG (0.5 mM) for 1 h. Each well (except for the control group) was subsequently treated with 1 mM MGO, and the cells were incubated for 24 h. Thereafter, 10 μM DCFH-DA was added, and the cells were incubated for 30 min. Intracellular ROS production was measured using a fluorescent microplate reader (Infinite M200; Tecan Austria GmbH) at 485 nm (excitation wavelength) and 530 nm (emission wavelength). The intracellular ROS production level was expressed as a percentage (%) of the decrease in fluorescence intensity relative to the untreated control (without sample or MGO).

### 3.9. Intracellular MGO Accumulation

The inhibitory effect of IPA on intracellular MGO accumulation was evaluated using an OxiSelect™ Methylglyoxal (MGO) Competitive ELISA Kit. Mesangial cells were seeded in a 6 well plate at a density of 1 × 10^6^/well, followed by incubation for 4 h. The cells were treated with IPA (0.2, 1, and 5 μM) and AG (0.5 mM) and incubated for 1 h. MGO was subsequently added, and the cells were cultured for an additional 24 h. The cells were collected using a scrapper, and intracellular MGO was quantified using the OxiSelect™ Methylglyoxal Competitive ELISA kit.

### 3.10. Immunofluorescence Analysis for Intracellular MGO-Derived AGE Accumulation

Immunofluorescence analysis was performed using an AGE antibody and 4,6-didiamidin-2-phenylindole (DAPI) to evaluate the effect of IPA on MGO-induced intracellular AGEs accumulation. The mesangial cells were distributed on a four-chamber slide at a density of 2 × 10^5^/chamber and incubated for 4 h. The cells were treated with IPA (0.2, 1, and 5 μM) and AG (0.5 mM), followed by incubation for 1 h. MGO was subsequently added, and the cells were cultured for an additional 24 h. They were fixed with 4% (*v/v*) formalin for 15 min and permeabilized with 0.1% (*v/v*) Triton X-100 for 5 min. After the removal of supernatant, the membranes were blocked for 30 min with 1% (*v/v*) BSA, followed by incubation with the AGE antibody (Abcam, 1:200) at 4 °C for 24 h. The cells were subsequently treated with the Alexa 488 secondary antibody at 24 °C for 2 h, followed by treatment with a mounting solution containing DAPI. Cells double-stained with AGEs and DAPI were examined for MGO-induced intracellular AGE accumulation using a fluorescence microscope (Zeiss Axio Observer A1, ZEISS, Jena, Germany).

### 3.11. Hoechst 33342/PI Double Staining

Hoechst 33342/PI double stanning was performed to evaluate the effects of IPA on MGO-induced renal cell apoptosis. The mesangial cells were seeded at a density of 5 × 10^5^ cells/well in 24-well plates and incubated for 4 h. The cells were treated with IPA (0.2, 1, and 5 μM) and AG (0.5 mM) and incubated for 1 h, followed by the addition of MGO and further culturing for an additional 24 h. The cells were immediately fixed with 4% (*v/v*) formalin for 10 min and permeabilized with 0.2% (*v/v*) Triton X-100 for 10 min. They were washed three times with PBS and stained with Hoechst 33258 (2 μg/mL) or PI (10 μg/mL) for 30 min at 37 °C. The cells stained with Hoechst 33258 or PI were observed for MGO-induced apoptosis or necrosis using a fluorescence microscope (Zeiss Axio Observer A1, ZEISS, Germany).

### 3.12. Apoptosis Analysis

MGO-induced renal apoptotic cell death was assessed using a Muse™ Annexin V & Dead Cell Kit (Luminex, TX, USA). Mesangial cells were seeded at 1 × 10^6^/well in a six-well plate, incubated for 4 h, and pretreated with different concentrations (0.2, 1, and 5 μM) of IPA and AG (0.5 mM) for 1 h. MGO was subsequently added to induce apoptosis, followed by incubation for 24 h. The cells were washed three times with cold PBS and incubated with the Muse™ Annexin V and Dead Cell reagent. The total number of apoptotic cells was measured using a MUSE flow cytometer (Merck Millipore, Sydney, Australia). The results were expressed as a percentage (%) of the total apoptotic cells (early and late) compared to the untreated control (without sample or MGO).

### 3.13. Western Blotting

Western blotting was performed to evaluate the effect of IPA on molecular mechanisms such as RAGE, apoptosis-related proteins, and Nrf2/ARE. The mesangial cells were seeded in a 100 mm petri dish (2 × 10^6^/dish) and incubated for 4 h. After the removal of the supernatant, the cells were pre-treated with IPA (0.2, 1, and 5 μM) or AG (0.5 mM) for 1 h. After 1 h, all wells (except for the control group) were treated with 1 mM MGO and incubated for 24 h. The cells were recovered using a scraper, and the protein was extracted using PRO-PREP™ Protein Extraction Solution. Total protein concentrations of the cell lysates were measured using a DC Protein Assay kit (Bio-Rad, Hercules, CA, USA). Equal amounts of diluted proteins were loaded onto a gel (Any kDa Mini-PROTEAN TGX Stain-Free Gel) and thereafter transferred onto PVDF membranes. The membranes were blocked with 5% (*v/w*) skim milk for 1 h. The membranes were subsequently incubated with primary antibodies (1:1000) at 4 °C for 24 h. Finally, the membranes were exposed to the WesternSure PREMIUM Chemiluminescent Substrate (LI-COR, Lincoln, NE, USA). Luminescence was detected using the ChemiDoc MP Imaging System (Bio-Rad, Hercules, CA, USA).

### 3.14. Statistical Analysis

All experiments were independently performed in triplicate, and data are presented as mean ± standard deviation (*n* = 3). To assess the significant differences in the mean values, a one-way analysis of variance (ANOVA) was performed, followed by the Tukey–Kramer honestly significant difference test (*p* < 0.05) using GraphPad Prism ver. 9 (GraphPad Software, Inc., San Diego, CA, USA).

## 4. Conclusions

MGO-derived AGEs have been shown to be important causative agents of several diabetic complications, including diabetic nephropathy. IPA, a phlorotannin compound isolated from the edible brown alga *Ishige okamurae*, showed natural AGEs inhibitory potential owing to its anti-glycation abilities. In addition, IPA exhibited a protective effect towards renal glomerular mesangial cells against MGO-induced oxidative stress through various mechanisms, including intracellular ROS scavenging, inhibition of intracellular MGO/MGO-derived AGEs accumulation, and inhibition of apoptotic renal cell death. Moreover, IPA was found to contribute to the renoprotective effect by suppressing RAGE protein expression and regulating the downstream apoptosis and Nrf2/ARE signaling pathways. Taken together, IPA has shown the potential to prevent kidney damage caused by MGO-derived AGEs through various pathways. These findings suggest that phlorotannin compounds present in seaweeds have the potential for development as naturally derived AGEs inhibitors, which would aid in the prevention and management of AGE-related diabetic nephropathy.

## Data Availability

The data presented in this study are available on request from the corresponding author.

## Supplementary Materials

The following supporting information can be downloaded at: https://www.mdpi.com/article/10.3390/md23010048/s1, Table S1: Antibody information used for western blot.
