# Supplementary material for: Ishophloroglucin A Isolated from Ishige okamurae Protects Glomerular Cells from Methylglyoxal-Induced Diacarbonyl Stress and Inhibits the Pathogenesis of Diabetic Nephropathy
Source: Mar Drugs. 2025 Jan 20;23(1):48. doi: 10.3390/md23010048 (PMC11766881; doi:10.3390/md23010048)

Supplementary Table S1. HPLC chromatogram of Ishophloroglucin A (IPA) isolated from *Ishige okamurae*.

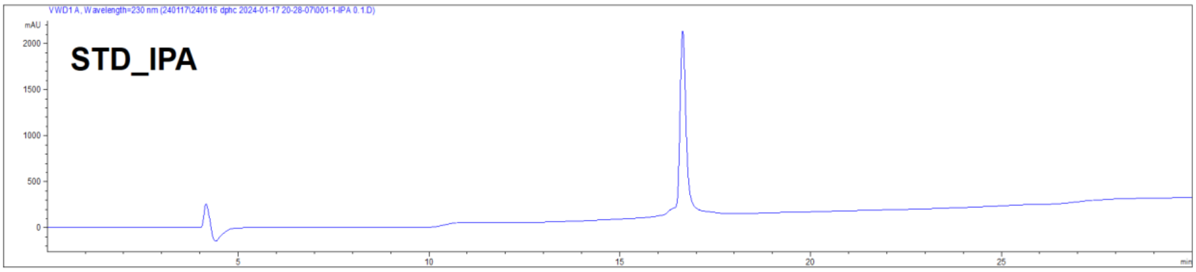

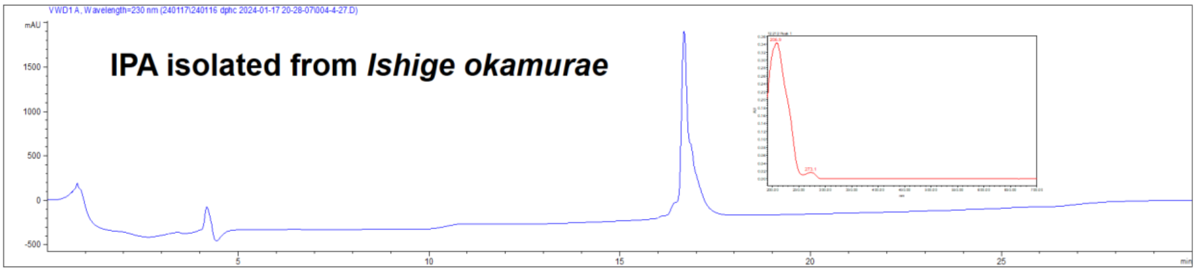

Supplement: Supplementary file 1 [file marinedrugs-23-00048-s001.zip › marinedrugs-3338164-supplementary.pdf]
